# Awareness of diabetes mellitus among diabetic patients in the Gambia: a strong case for health education and promotion

**DOI:** 10.1186/1471-2458-13-1124

**Published:** 2013-12-05

**Authors:** Mafomekong Ayuk Foma, Yauba Saidu, Semeeh Akinwale Omoleke, James Jafali

**Affiliations:** 1 School of Medicine and Allied Health Sciences, University of The Gambia, Banjul, The Gambia, West Africa; 2 Medical Research Council, The Gambia Unit, Fajara, The Gambia, West Africa

**Keywords:** Diabetes mellitus, Awareness, The Gambia, Causes, Prevention and management, Health education and promotion

## Abstract

**Background:**

Awareness of various aspects of Diabetes Mellitus (DM) is essential for the prevention, management and control of the disease. However, several studies have consistently shown that awareness of DM in the general population is low. None of these studies, however, was conducted in The Gambia, even though the condition constitutes a major public health problem in the country. In this paper, we assessed the awareness of DM among diabetic patients attending the Medical Out-Patient Department (MOPD) of Royal Victoria Teaching Hospital (RVTH), Banjul.

**Methods:**

We interviewed 200 patients attending the MOPD of RVTH. We used a tool containing questions on patient’s demographic characteristics and awareness of various aspects of DM including general knowledge on DM, causes, complications, management and prevention.

**Results:**

Of the 199 patients who were aware of their condition, only 47% said they knew what DM is. Similarly, 53% of the study participants had no knowledge of the causes of DM and about 50% were not aware of the methods of prevention. 67% knew that DM can result to loss of sight while 46.5% knew that DM can cause poor wound healing. Few respondents knew that DM can lead to kidney failure (13.5%), skin sepsis (12.0%), heart failure (5.5%) and stroke (4.5%). Close to 50% of the respondent did not know how DM can be prevented. Level of education, duration of illness and knowledge of a family member with diabetes were important predictors of knowledge in our study.

**Conclusion:**

Our study shows that the majority of patients attending the MOPD have poor knowledge on several aspects of DM. Hence, there is need for conscious efforts towards improving the level of awareness through health education and promotion, not limited to the hospital but also within the general population, as part of strategies to prevent, manage and control DM.

## Background

Diabetes Mellitus (DM) has emerged as one of the most challenging public health problems in the 21^st^ century. It currently affects over 366 million people worldwide and this figure is likely to double by 2030 [1,2]. The greatest burden of this condition is felt in low and middle-income countries, and these nations account for about 80% of all cases of diabetes [3]. In sub-Saharan Africa alone, there are about 12 million people suffering from this condition and there are projections that this number will reach 18 million by 2030, making the region the one with the fastest growing rates of diabetes mellitus in the world [2,4]. In the Gambia for instance, the incidence of DM has been projected to increase by three-fold within this period; that is from 22,000 cases in 2012 to 61,000 by 2030 [5].

This silent, but imminent, public health problem would impose substantial challenges on the healthcare systems as well as on the economy of most developing nations in the near future. This is because a significant proportion of individuals who suffer from the condition in these countries are within the reproductive age [1,6]. These are the same individuals who are expected to drive the economic machinery in these nations so as to achieve the agreed millennium development goals [7]. When the disease affects these individuals, and if not properly controlled, it may lead to lifelong complications, which are generally associated with increased morbidity and mortality [8,9]. For instance, poorly controlled DM can cause damage to eyes (leading to blindness), kidneys (leading to renal failure), and nerves (leading to impotence and foot disorders/possibly amputation) as well as increased risk of heart disease, stroke, and poor blood supply to the limbs [9]. Most of these complications are not only irreversible, but there are also costly to manage as they generally require management in specialized centers with sophisticated infrastructure and equipment, well trained staff and potent medications, which are all scarce in SSA [10].

Since most of these specialized centers are not available in many SSA settings, patient education becomes a central component in the prevention and control of this disease in SSA [11,12]. Such education should lead to diet modification, increased physical exercise and lifestyle changes including the promotion of weight loss [12,13]. These educational programs should help people assess their risks of diabetes, motivate them to seek proper treatment and care and inspire them to take charge of their disease [7,13]. In addition, it should enable early detection and treatment of complications as well as enhanced early referrals of cases to specialized centers for management and follow-up.

Although the importance of educational programs in the prevention and control of DM is well recognized [14], there are concerns whether these programs are achieving the desired goal of increasing awareness of DM in developing countries. Indeed, several studies have consistently shown that awareness of the DM in the general population seems to be low [7,15-23]. For example, Ulvi et al., showed that a significant number of people in rural Islamabad had little or no knowledge of DM, and even the few who claimed to be aware of the condition only knew it by the name “sugar” and had never heard the term “Diabetes Mellitus” [15]. Similarly, others have shown that many people are still ignorant about several aspects of the disease as well as approaches that are necessary for the prevention and control of DM [16,17].

While the awareness of DM has been documented in many developing countries, very few studies have been done in SSA [7,18,19,21,22]. None of these studies, however, was conducted in The Gambia, even though the condition constitutes a major public health problem in the country [5]. Consequently, this study sets out to assess the awareness of DM among patients attending the Medical Out-Patient Department (MOPD) of Royal Victoria Teaching Hospital (RVTH), Banjul.

## Methods

### Study setting

The study was conducted in The Gambia, a small West African country that is completely surrounded by Senegal except for a small coastline in the west. The country is a narrow strip of land of about 30–50 kilometers wide and about 350 kilometers long. It has a population of about 1.7 million inhabitants, 22,000 of whom are known diabetics. Many of these patients receive care at the RVTH, which is the only tertiary health institution in the country. RVTH is located in the capital city, Banjul, and has a capacity of 540 beds. The institution runs several MOPD clinics, including the diabetic clinic which holds on Wednesdays.

### Procedures

Our study participants were randomly selected from a pool of patients attending weekly diabetic clinics at the MOPD of RVTH, Banjul. We interviewed a total of 200 patients from October - December 2012. Briefly, the study was explained to all patients attending the facility during the study period by one of the researchers and two trained nurses working at the MOPD. Participants who agreed to participate were requested to provide consent by signing or thumb printing on a consent form. A two- page questionnaire was administered to the study participants. The questionnaire contained series of questions on participant’s demographic characteristics and awareness of DM including general knowledge on DM, causes, complications, management and prevention. The questionnaires were interpreted into local languages, to those who could not understand or read English by trained staff.

### Data analysis

Filled questionnaires were reviewed for completeness and accuracy before data entry. Data were doubled entered in EPI info version 7.0 (CDC Atlanta) and exported to Stata, version 12.1 (StataCorp LP, College Station, Texas, USA) for analysis. Awareness of the different aspects of DM was estimated using summary statistics. In addition, we assessed the effect of independent (exposure) variables (such as age, education, ethnicity, occupation, place of residence and gender and co-morbidities) on awareness of DM. To this end, logistic regression models were applied to estimate Odds ratios and their 95% confidence intervals while mutually adjusting for the confounding effects of other factors under investigation. In all the regression models, the outcome variable of interest was awareness of DM, which was defined as knowledge of the following aspects of DM; definition, causes, complications, management and prevention. All tests were conducted at an alpha level of 5% and hence, any p-value of less than 0.05 was considered as a significant association. Results are presented in tables, graphs and text.

### Ethical aspects

This study was reviewed and approved by the Ethical and Research Committee of RVTH. Informed consent was obtained from all participants and the data collected were kept confidential. No names were mentioned on the questionnaires.

## Results

### Baseline characteristics of study population

Overall, a total of 200 adults provided consent and were interviewed. The median age of the study population was 53 years (range 18–80). The socio-demographic characteristics of these participants are presented in Table 1. Fifty nine percent of the study participants were females. The predominant ethnic group was Mandinka (33.5%), followed by Wolof (24%) and then Fula (21.5%). Over 9 in 10 of the study population were Muslims and about a third of them have attended Arabic school. A significant proportion of these participants was married (74.5%) and was essentially residing in urban areas (82%), notably the Greater Banjul area and Kanifing Municipality. Over 80% of these participants had a non-sedentary occupation such as farming, fishing, and carpentry amongst others.

**Table 1:** Socio-demographic characteristics of study population

**Variable**	**n (%)**	**95% CI**
Sex		
Females	118 (59)	51.84- 65.89
Males	82 (41)	31.11-48.16
Education		
None	35(17.5)	12.50-23.49
Arabic	72(36)	29.35-43.07
Primary school	21(10.50)	6.62-15.60
Middle school	30(15.00)	10.35-20.72
High school	29(14.50)	9.93-20.16
University	13(6.50)	3.15-10.86
Marital status		
Married	149 (74.5)	67.89-80.39
Single	14 (7.0)	3.88-11.47
Separated	6 (3.0)	1.11-6.42
Divorced	13 (6.5)	3.51-10.86
Widowed	18 (9.0)	5.42-13.85
Area of residence		
Urban	163 (81.5)	75.62-86.72
Rural	37 (18.5)	11.47-22.80
Ethnicity		
Mandinka	67 (33.5)	27.0-40.50
Wolof	48 (24.0)	18.26-30.53
Fula	43 (21.5)	16.02-27.85
Sarahule	9 (4.5)	2.08-8.35
Aku	7 (3.5)	1.42-7.08
Manjago	3 (1.5)	0.31-4.32
Others	23 (11.5)	7.42-16.75
Religion		
Islam	185 (92.5)	87.93-95.74
Christian	15 (7.5)	4.26-12.07
Occupation		
Non-sedentary	165 (83.5)	76.12-87.25
Sedentary	33 (16.5)	12.29-23.24
Life style		
Non-sedentary	161(80.50)	74.32-85.75
sedentary	39 (19.50)	14.25-25.68
Residence		
Urban	164 (82)	75.96-87.06
Rural	36 (18)	12.94-24.04

**Figure 1:**
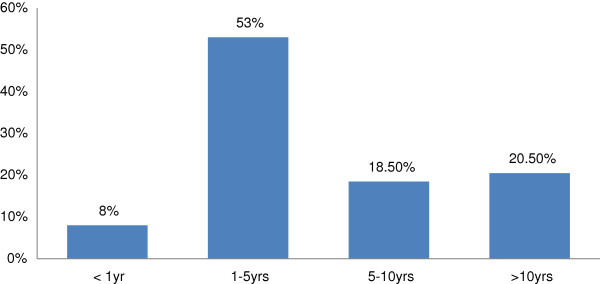
Duration of illness since diagnosis of diabetes was established.

**Figure 2:**
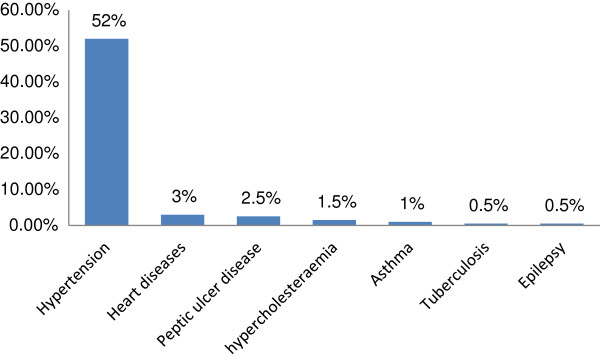
Concurrent morbidities with Diabetes Mellitus.

Over half of the study population has been diabetic for a period between one to five years (Figure 1) and 52% of them were known hypertensive (Figure 2). Approximately 80% of these participates knew someone who was diabetic in the family and most participants had a sibling (26.3%), mother (18.8%), father (16.3%) or grandparents (3.1%) in the family who were diabetic (Table 2).

**Table 2:** Knowledge of someone with Diabetes

**Variable**	**n (%)**	**95% CI**
Participant has/had a family member who is/was diabetic		
No	41 (20.50%)	15.13-26.77%
Yes	159 (79.50%)	73.23-84.87%
Relationship of diabetic person to participant		
Father	26 (16.25%)	10.90-22.90%
Grandparents	5 (3.13%)	1.02-7.14%
Mother	30 (18.75%)	13.02-25.67%
Sibling	42 (26.25%)	19.62-33.78%

### Knowledge of subject matter

Of the 200 participants, only one was not aware that he was diabetic even though he was a known hypertensive (Table 3). Of the participants who were aware of their condition, only 47% said they knew what DM is (Table 3). Similarly, a significant proportion of them were not aware of the actual cause of DM. For instance, 53% of the study participants had no knowledge of the causes of DM while 16% said that it could be caused by high sugar intake. However, some respondents knew one cause of DM. For instance, 27% of the participants said the condition runs in the family while others said DM can be caused by insufficient insulin production (6.5%) or poor insulin utilization (2.0%).

**Table 3:** Knowledge of DM by participants

**Knowledge of DM**	**n (%)**	**95% CI**
Knew that they were diabetic		
Yes	199 (99.5)	97.3-99.99
No	1 (0.5)	0.01%-2.75
Said they knew what diabetes is		
Yes	94 (47)	39.92-54.17
No	106 (53)	45.83-60.08
Knowledge of the cause of DM		
I don’t know	106 (53.0)	45.85-60.08
Family history	54 (27.0)	20.98-33.72
High sugar intake	32 (16)	11.21-21.83
Lack of insulin	13 (6.5)	3.51-10.83
Failure of body to use insulin	4 (2.0)	0.55-5.04
Others	3 (1.5)	0.31-4.32
Knowledge of complications of DM		
Loss of vision	134 (67.0)	60.02-73.47
Poor wound healing	93 (46.5)	39.44-53.67
Amputations	62 (31.0)	24.67-37.91
Kidney failure	27 (13.5)	9.09-19.03
Skin sepsis	24 (12.0)	7.84-17.33
Heart failure	11 (5.5)	2.78-9.63
Stroke	9 (4.5)	2.08-8.37
Knowledge of the management of DM		
Diet and medication	134 (67.0)	60.02-73.47
Diet, Exercise and medication	57 (28.5)	22.36-35.29
Diet and Exercise	4 (2.0)	0.55-5.04
Medication	4 (2.0)	0.55-5.04
Diet	1 (0.5)	0.01-2.75
Knowledge of preventive measures		
I don’t know	90 (45.7)	38.59-52.91
Healthy diet	67 (34.0)	27.43-41.08
Eating less sugar	28 (14.2)	9.66-19.88
Physical activity	11 (5.6)	2.82-9.77
Weight loss	1 (0.5)	0.01-2.80

Knowledge of the visible complications of DM appeared to be somewhat better than knowledge of other complications. For example, 67% knew that DM can result to loss of sight while 46.5% knew that DM can be associated with poor wound healing. In addition, about a third knew that DM can result to amputations (Table 2). Even though the majority of the participants could identify the visible complication, awareness of the invisible complications of DM was comparatively lower. For instance, few of these participants knew that DM can lead to kidney failure (13.5%), skin sepsis (12.0%), heart failure (5.5%) and stroke (4.5%).

Regarding the management of DM, a significant proportion (67%) of these participants said DM can be managed by dietary modification and medication while 28.5% felt that the condition can be managed by a combination of dietary modification, exercise and medication. Only a few said that dietary modification (0.5%) or medications (2.0%) alone are the mainstream of diabetic management (Table 2).

Like other aspects of DM, knowledge of the preventive measures was poor. Indeed, close to 50% of the respondent did not know how DM can be prevented. About a third of our study participants knew that a healthy diet was essential for the prevention while about 6% said that DM could be prevented by physical activity.

### Association between awareness of DM and some selected variables

Table 4 presents the association between awareness of DM and some selected variables such as age, education, ethnicity, occupation, place of residence and gender. As illustrated, educational level was an important predictor of awareness. For instance, participants with university education were more likely to be aware of DM than those with no formal education and this difference was statistically significant (aOR = 10.4; p = 0.03). The same was true for participants with middle (aOR = 5.2; p = <0.01) and high school education (aOR = 19.8; p = <0.01). Secondly, participants who knew someone with diabetes were more likely to be aware of DM than those who did not (aOR = 2.8; p = 0.01). There was also a positive association between the duration of illness and awareness of DM. For instance, participants who had been living with this condition for more than ten years were more likely to be aware of DM than those who had been living with the condition for less than one year (aOR = 3.8; p = 0.04). However, no significant difference was observed between participants who had been living with the condition for less than five years (p = 0.15). Likewise, no significant association was found between those who regularly attended their monthly clinic visits and those who did not (aOR = 1.18; p = 0.82). And finally, there was also no statistically significant association between awareness of DM and variables, such as age, ethnicity, sex, occupation, place of residence and co-morbidities.

**Table 4:** Odds ratios and their 95% confidence interval for the associations of awareness of DM with various social demographic factors and co-morbidities

**Variable (reference)**	Adjusted Odds Ratio*	**95% C.I.**	**P-Value**
Age of respondent (< 45 years)			
45-60 years	1.02	0.98-1.05	0.32
> 60 years	3.60	0.94-13.70	0.06
Education level (Arabic school)			
High school	19.77	5.19-75.19	<0.001
Middle school	5.23	1.75-15.58	0.0030
None	1.37	0.47-3.94	0.5630
Primary	1.48	0.47-4.65	0.5022
University	10.40	2.23-48.49	0.0029
Ethnicity (Aku)			
Fula	19.07	0.85-425.26	0.0628
Mandinka	13.53	0.59-312.18	0.1038
Others	5.36	0.32-90.69	0.2450
Sarahule	3.08	0.07-144.20	0.5663
Wolof	18.93	0.86-418.63	0.0627
Occupation ( Non sedentary)			
Sedentary	1.91	0.75-4.90	0.1775
Place of residence ( Rural )			
Urban	1.35	0.55-3.33	0.5176
Sex (Female)			
Male	1.23	0.55-2.72	0.6272
Religion (Christianity)			
Islam	0.21	0.02-1.92	0.1656
Adherence to monthly clinic visits (No)			
Yes	1.19	0.26-5.46	0.8233
Duration of DM illness (<1 year )			
>10 years	3.83	1.06-13.90	0.0409
5-10 years	3.53	0.96-12.99	0.0578
1-5 years	2.39	0.72-7.89	0.1527
Knowledge of someone with diabetes (No)			
Yes	2.78	0.67-2.24	0.0092
Co-morbidities with DM			
Hypertension (Yes vs. No)	1.01	0.58-1.76	0.9729
Heart Disease (Yes vs. No)	2.31	0.41-12.91	0.3400
Hypercholesterolemia (Yes vs. No)	1.01	0.58-1.76	0.9729

* Mutually adjusted for other variables shown in the table.

## Discussion

This study was undertaken to evaluate the awareness of DM among diabetics attending the MOPD at the RVTH as well as to determine the predictors of awareness/ knowledge of DM. The specific objectives were to assess how knowledgeable our study participants were regarding the definition of diabetes, its causes, management and complications as well as its prevention. Our findings show that the general awareness of these aspects is low among diabetic patients in The Gambia. Indeed, over half of our study population was unaware of what DM was. This finding correlates with that of Muninarayana *et al.* who reported that 50% of diabetic patients in Tamaka Kolar (India) had no knowledge of diabetes [16]. Similar findings have also been reported from Kenya [7].

Knowledge of the causes of diabetes was also low. Few participants (mostly health workers) knew that the condition could be caused by lack of insulin (7%) or failure of the body to use insulin (2%). This finding is in agreement with that of Unadike *et al*. who reported that only few respondents in Uyo (Nigeria), knew that lack of insulin can cause diabetes [19]. It is striking to discover that about 80% of our study participants knew a family member who was diabetic; however, only about a third of them knew that diabetes could be familial. This finding is consistent with that of Hashmi *et al.*, who reported that most patients in Lohore (India) were unaware that diabetes runs in the family [23]. A significant proportion of our study participants felt that diabetes can be caused by high sugar intake or other factors such as hypertension and stress, a belief that can only be altered if these patients are provided with appropriate education regarding the causes of diabetes.

Knowledge of the visible complications of DM such as loss of vision, poor wound healing and amputations appeared to be somewhat better than knowledge of non-visible complications such as heart failure, kidney failure and stroke. This observation is consistent with findings reported by Unadike *et al.*[19] and Muninarayana *et al*. [16]. In our study, only patients who were suffering from kidney and heart failure said that these complications were as a result of DM. This finding suggests that patient education on the complications of DM seems not to be optimal at the MOPD of RVTH. There is therefore the need to educate patients on these complications as this may encourage them to adopt appropriate measures that may be vital in managing the disease in order to prevent these complications.

Most complications of diabetes can be prevented via dietary modification, exercise, changes in lifestyle, and the use of anti-diabetic medications for individuals who are unable to achieve a suitable glycemic level with non-pharmacological methods [9]. We noted a marked variance in awareness of these aspects of diabetes management among our study participants. For instance, while over 90% knew that diabetes can be managed with dietary modification and drugs, only about a third (mostly males) of our study participants knew that exercise is an essential component of diabetes management. Our finding is in line with a popular belief in the general population that dietary adjustment and medications are the main stay in the management of diabetes. The lack of awareness of the importance of exercise in the management of diabetes is not a surprising finding, particularly in a predominantly Muslim population, and this is consistent with the findings of Baskin *et al.* in rural Tanzania [24].

Knowledge of how diabetes can be prevented was also poor. Indeed, almost half of our study participants had no clue on how the condition can be prevented while a very small number thought that weight loss (0.5%) and exercise (5.6%) were important measures in preventing the condition. Similar observations have been reported from India [23], Oman [17] and Tanzania [24]. These findings are worrisome, particularly for a country like The Gambia, whose prevalence of DM is expected to triple between now and 2030 [5]. There is therefore a need for more educational campaigns to promote modification of lifestyles as well as adherence to exercise and dietary prescription. Such campaigns should be simplified to enable individuals with low educational status to understand the messages.

Three important predictors of awareness were identified in our study. First, those with formal education beyond middle school had a better understanding of all aspects of the DM than those with Arabic or no education at all. This finding, which is consistent with that of Muninarayana et *al.,*[16], may essentially be explained by the fact that those with formal education beyond middle school might have learnt about DM from their schools or are more likely to access the internet or magazines/ books. The second predictor of awareness was knowledge of a family member with diabetes. Indeed, participants who had a family member with this condition were likely to be aware of the condition that those who did not (p = 0.01). Most of these participants who had a family member with diabetes acknowledge that they had, at one point in time, accompanied their diabetic relatives to the clinic, assisted in their care or stay with them in the hospitals. This experience could have given them a greater familiarity with the symptoms, causes, management and preventive measures for this illness. Third, there was a significant association between the duration of illness and awareness of diabetes. Those who have been living with this disease for more than ten years were more knowledgeable than those who have been living with the condition for less than one year (p = 0.04). However, no significant difference was found between those who have been living with the condition for 5–10 years and less than1year (p = 0.06). This finding seems to suggest that there is no proper diabetic health education and promotion at the MOPD. This interpretation is further strengthened by the fact that no statistically significant difference was observed between those who attended the clinic regularly and those who did not (p = 0.82). Indeed, during our study period (October-December 2012) no lectures/health talks were held at the facility. The apparent absence of a proper health education and promotion package may be due to insufficient training of health workers, limited staff strength and little priority that is being accorded to this condition and other non-communicable diseases. This situation, however, needs to be redressed; given that diabetes is an important cause of mortality and morbidity, there is a need to institute or re-enforce patient health education lectures during diabetic clinics at the MOPD. Similarly, since the MOPD is the only reference out-patient medical facility in the Gambia, it is important for these public health promotion and prevention campaigns to be extended beyond hospital settings. In other words, a nationwide diabetic education program should be designed and effectively implemented at both community and hospital settings across the nation.

Although this study has provided useful information about the state of awareness of DM among diabetic patients attending the lone reference out-patient medical facility in The Gambia, certain limitations must be acknowledged. As the majority of our study participants had no formal education, accurate administration of our questionnaires (written in English) depended on the translation of the interviewer, which could have in some way introduced a translation bias. Similarly, the documentation of responses also depended on the interviewer’s understanding of the response, which could have also been subjected to bias or misrepresentation. Secondly, responses to most questions (e.g. duration of illness, co-morbidities etc.) were self-reported and no references was made to medical reports/charts as these documents were hardly available. This reliance on self-reporting may be prone to “recall bias”. Third, the source of patient information about DM was not included in this study and this makes it difficult to suggest an appropriate channel through which information can be delivered. Additionally, our study did not seek to investigate the differences in awareness among participants suffering from the major types of DM. And finally, the study essentially focused on patients attending the MOPD, the majority of who were resident in urban areas. This limitation implies that the findings of this study may not be generalizeable to the entire country. Nonetheless, the present findings lay the groundwork for further similar studies in other parts of the country.

Given that DM is emerging as a major public health challenge in The Gambia and that the current health infrastructure is inadequate to address this challenge, effective control and prevention strategies based on sound educational programs need to be defined and implemented [12]. Those living with this condition should be properly educated on lifestyle changes and diet modifications so as to prevent lifelong complications. These programs should also target community and religious leaders as well as other social groups (including schools) because the impact of this disease is felt by the entire population [12]. This information can be disseminated via a variety of channels including radio and television shows, newspapers, automated mobile phone messages, internet and formal group talks. Most of this information (particular radio and TV shows) should be delivered in local languages since the majority of the population does not have formal education. Additionally, health professionals need to be thoroughly trained so that they can effectively educate their patients. Furthermore, diabetic or preferably education on chronic non-communicable diseases should also be introduced in school curriculum. Investing on health education might lead to a substantial benefit to the state as this would reduce the cost of healthcare (which is currently being subsidized by the state) or economic loss through job absenteeism following chronic morbidity associated with the disease. And finally, given that about 4 in 5 of our study population had a family member who was diabetic, targeted screening should be done on family members of all diabetic patients.

## Conclusion

Diabetes mellitus poses a major health challenge both epidemiologically and economically in The Gambia and Africa in general. However, awareness of this pathological condition among diabetics is low in many African settings, let alone the general population. Our study shows that the majority of patients attending the MOPD have poor knowledge on several aspects of the condition including its causes, complications, management and prevention. Hence there is an urgent need to raise the level of awareness of this silent but deadly disease condition in the Gambian population.

## Competing interests

The authors declare no conflict of interest in the design, conduct and reporting of the findings of this study.

## Authors’ contributions

FMA participated in the design of the study and acquisition of the data. SY participated in the design of the study, developed the study tools, participated in the data analysis and wrote the first draft of the manuscript. OSA participated in the design of the study and its coordination as well as proof-read all the drafts. JJ led the data analysis process. All authors read and approved the final manuscript.

## Pre-publication history

The pre-publication history for this paper can be accessed here:

http://www.biomedcentral.com/1471-2458/13/1124/prepub
